# Evolution of total body and regional adiposity from late adolescence to early adulthood in a birth cohort study

**DOI:** 10.1186/s12986-019-0347-6

**Published:** 2019-03-25

**Authors:** Silvana Paiva Orlandi, Leonardo Pozza Santos, Ana Maria Baptista Menezes, Fernando C. Wehrmeister, Helen Gonçalves, Maria Cecília Formoso Assunção

**Affiliations:** 1 Faculdade de Nutrição, Nutrition College, Federal University of Pelotas, Rua Gomes Carneiro, 01 – 2 piso, Pelotas, 96010-610 Brazil; 2 Post-graduate Program in Epidemiology, Federal University of Pelotas, Pelotas, Brazil; 3 Nutrition College, Federal University of Pampa, Itaqui, Brazil

**Keywords:** Body composition, Body shape, Cohort studies

## Abstract

**Background:**

Differences in total body and regional adiposity according to sex are observed from an early age, but these differences become more evident after puberty due to hormonal changes. We aimed to assess the evolution of total body and regional adiposity from 18 to 22 years of age and the associated sociodemographic and nutritional characteristics.

**Methods:**

In total, 3274 individuals from the 1993 Pelotas birth cohort study followed up at 18 and 22 years of age. Measures of total body and regional adiposity were assessed using whole-body dual-energy X-ray absorptiometry (DXA) and the TC2 Three-Dimensional Photonic Scanner. We used fat mass index obtained from DXA as a measure of total body adiposity, and android and gynoid fat mass percentages (android or gynoid fat mass [kg]/total fat mass [kg])*100) as measures of regional adiposity. In addition, waist, hip and thigh circumferences from the photonic scanner were also used as measures of regional adiposity. We evaluated these measurements at 18 and 22 years of age by sex and estimated differences between them according to sociodemographic and nutritional characteristics.

**Results:**

While men and women did not differ in terms of BMI, females exhibited a higher fat mass index, gynoid fat mass percentage, and hip and thigh circumferences; men exhibited higher android fat mass percentage and waist circumference at both time points. Increases in all body measurements from age 18 to 22 were observed in men and women, except for gynoid fat mass percentage, which decreased in both sexes. Socioeconomic position and race were the independent variables most associated with adiposity rising from age 18 to 22 in women, with black women and women of lower socioeconomic positions exhibiting larger increases in adiposity.

**Conclusion:**

There was an increase in adiposity and a centralization of body shape from late adolescence to early adulthood, indicating possible early risks for noncommunicable diseases in this cohort.

## Introduction

High prevalence of overweight and obesity are important public health concerns worldwide, representing a risk factor for several diseases [1, 2]. Children and adolescents have been experiencing increases in overweight and obesity rates, mainly in low- and middle-income countries [1, 3]. In Brazil, nationally representative studies have shown a rise in the proportion of young people diagnosed as overweight or obese, reaching 20% of adolescents in 2009 [4].

Obesity in adolescence may be assessed through body mass index (BMI)-for-age by comparison to the World Health Organization (WHO) 2007 reference [5]. However, BMI has known limitations, as it does not distinguish body composition. In contrast to BMI, several body composition methods can define the extent to which body weight is composed of fat mass and lean mass. However, it is important to look beyond overall body adiposity and take the distribution of body fat into account, since investigations have shown independent roles of different types of fat depots in disease risks and mortality [6–10].

Although men and women have similar average BMIs in adolescence and early adulthood [11, 12], differences in total body and regional adiposity according to sex are observed from an early age [13]. However, these differences become more evident after puberty due to hormonal changes, with girls showing higher fat mass and boys showing a more centralized body shape [14, 15].

From the 1993 Pelotas (Brazil) birth cohort study we have data on total body and regional adiposity from participants who followed-up when they were 18 and 22 years of age. This information may enable us to better understand the amount and distribution of body fat in a population-based sample. Therefore, we aimed to assess the evolution of total body and regional adiposity from 18 to 22 years of age and compare this evolution among socioeconomic, demographic and nutritional characteristics.

## Methods

### Subjects

Pelotas is a southern Brazilian city with more than 330,000 inhabitants, according to the last national census in 2010. It is located 120 km away from the Uruguayan border and has a lower gross domestic product but higher human development index when compared to Brazil as a whole.

In 1993, a second birth cohort study was launched in Pelotas, where five maternity hospitals were visited and all births were monitored. At that time, the research team invited mothers who gave a live birth in these maternity hospitals to take part in the study, and those mothers who agreed to participate were interviewed in the first 24 h after delivery. Less than 1% of mothers refused to participate, and 5249 babies were included in the study.

Several follow-ups of this cohort study have been performed since birth. In all visits before the cohort members had reached 11 years of age, only subsamples were sought. All cohort members were sought when they were 11, 15, 18 and 22 years old. In all follow-up visits, the retention rates were greater than 75%. Trained field workers collected information about anthropometry, nutritional status, cognitive development and socioeconomic position (SEP) in all follow-ups. Details of the perinatal and subsequent follow-ups were reported previously [16, 17].

### Total body and regional adiposity

At ages 18 and 22 years of age, participants received an invitation to visit the study clinic. These visits lasted approximately four hours, and interviewers collected information about anthropometry (i.e., weight and height), body composition, body shape, lifestyle, feeding habits, and health status. Some details about the methods involved in these follow-ups were published previously [17].

Measurements of total body and regional adiposity included in this study were collected using whole-body dual-energy X-ray absorptiometry (DXA) (GE Lunar Prodigy densitometer) and the TC2 Three-Dimensional Photonic Scanner (North Carolina, USA; www.tc2.com). For DXA examination, participants remained barefoot and in a supine position, and wore light and tightly fitted clothes. Field workers assessed the quality of DXA exams and repeated measurements when necessary. DXA equipment was calibrated in the beginning of each working day according to the manufacturer. Pregnant women, women who had a suspicion of being pregnant and individuals who did not fit entirely in the scanning area (height > 1.90 m, weight > 120 kg, body width > 60 cm) were not included in this examination.

In the Photonic Scanner assessment, participants remained in a standing position wearing the same clothes used for DXA examination and underwent two photonic scans. If the difference in waist circumference was greater than 10 mm, the technician carried out extra body scans until two measurements of waist circumference with a difference lower than 10 mm were obtained. The arithmetic mean of these two measurements was then calculated. The TC2 Photonic Scanner was also calibrated at the beginning of each working day, and a trained technician assessed the quality of body scans generated by the scanner. Confirmed and suspected pregnancy were also exclusion criteria for the Photonic Scanner assessment.

Height and weight were also collected in the clinic. Height was measured twice using a Harpenden metal stadiometer (Holtain, Crymych, UK) with 1 mm of precision. Weight was measured using a high precision scale (0.001 kg) as part of the BodPod machine (Cosmed, Italy, http://goo.gl/7jzfLc). All measurements were collected on the same day, and the field workers who carried out anthropometric measurements were standardized based on Habicht criteria [18].

We selected body mass index (BMI) as well as fat mass index as measures of total body adiposity. BMI was calculated by dividing weight (kg) by height (m^2^), while fat mass index was calculated by dividing total fat mass (kg) from DXA by height (m^2^).

To assess regional adiposity, we used measures of regional fat mass from DXA, which indicated the distribution of central and peripheral fat mass (i.e., android and gynoid fat mass percentages). The percentages of android and gynoid fat mass were calculated as follows: (android or gynoid fat mass [kg]/total fat mass [kg])*100.

In the regional adiposity assessment, we also used body circumferences calculated by the Photonic Scanner. We selected waist circumference (cm) to represent central adiposity and hip and thigh circumferences (cm) to represent peripheral adiposity.

### Independent variables

To assess the socioeconomic, demographic and nutritional characteristics associated with the evolution of adiposity, we added information collected in the perinatal study as well as from the follow-up at 18 years of age. We used the SEP at birth and at 18 years of age. The SEP at birth was calculated according to the Brazilian minimum wage in 1993. The SEP at 18 years of age was measured from a quintiles of assets index based on household assets [19]. We also used information about maternal age at birth, pregestational maternal BMI, birthweight, self-reported race and daily energy intake at 18 years of age. Daily energy intake at 18 years of age was calculated using the total amount of kilocalories consumed per day according to information collected with a Food Frequency Questionnaire (FFQ). We calculated the ratio between energy intake and total energy expenditure in order to detect overestimated daily energy intake. The results were standardized, and those 2 standard deviations (SD) below and 2 SD above the mean were considered outliers and excluded from our analyses. The FFQ applied in the follow-up at 18 years of age was created based on a previous FFQ used for the 1993 and 1982 cohort studies and included 88 food items with closed responses related to portion size [20].

### Statistical analyses

We used means and standard deviations to describe measures of total body and regional adiposity at 18 and 22 years of age stratified by sex. As one of the assumptions of an analysis of variance (ANOVA) test is independence of observations and body composition measurements collected at 18 and 22 years of age were dependent, we used a repeated-measures ANOVA to assess the association between these measures and sex. Additionally, we also checked second order interactions between sex and age to evaluate whether the evolution in measures of total body and regional adiposity between the two follow-ups varied according to sex. A significant interaction between sex and age indicated sex differences in the evolution of adiposity between the two follow-ups.

To analyze the socioeconomic, demographic and nutritional characteristics associated with the evolution of adiposity from 18 to 22 years of age, we first calculated the difference between these measures by subtracting values at 22 years of age from those at 18 years of age. Afterward, we performed a linear regression analysis of these differences according to socioeconomic position at 18 years of age (i.e., respective quintiles), maternal pregestational BMI (i.e., normal, overweight, obese), maternal age at birth (i.e., < 18 years, 18 to 35 years and > 35 years), birth weight (i.e., < 2500 g, 2500 to 4000 g and > 4000 g), race (i.e., white, brown and black according to the classification used by the Brazilian Institute of Geography and Statistics) and daily energy intake at 18 years of age (i.e., respective quintiles).

As SEP at birth is associated with all measurements of adiposity as well as with sociodemographic characteristics, analyses aiming to check the sociodemographic and nutritional characteristics associated with adiposity evolution were adjusted for SEP at birth. All analyses were stratified by sex and performed using Stata 13.0 (Stata Corp., College Station, Texas, USA).

## Results

We obtained information from 3274 individuals who followed-up at 18 and 22 years of age with available data for total body and regional adiposity. Women represented 52% of the sample. Approximately ¼ of the total sample was born to overweight or obese mothers, and 7.8% of the sample were born to adolescent mothers. Almost 10% had low birth weight, and 1/3 were brown or black individuals (Table 1). The mean daily energy intake at 18 years of age was 3064 kcal, after the exclusion of overestimated values (data not shown in tables).

**Table 1:** Socioeconomic, maternal and individual’s characteristics for those who were followed at 18 and 22 years. The 1993 Pelotas Cohort Study (*N* = 3274)

Characteristics	Followed at 18 and 22 years
	N (%)
Sex	
Men	1584 (48.4)
Women	1690 (51.6)
Pre-gestational maternal BMI (Kg/m^2^)	
Normal	2447 (76.8)
Overweight	583 (18.3)
Obese	156 (4.9)
Maternal age at birth (years)	
< 18	256 (7.8)
18–35	2709 (83.0)
> 35	300 (9.2)
Birth weight (g)	
< 2500	294 (9.0)
2500–4000	2798 (85.8)
> 4000	171 (5.2)
Skin colour	
White	2015 (66.0)
Brown	484 (18.9)
Black	554 (18.2)

Maximum percentage of unknown observation: 221 (6.8%) for skin colour

Both men and women showed increases in BMI from adolescence to adulthood (from 23.3 to 25 kg/m^2^ in men and from 23.4 to 25.4 kg/m^2^ in women), but they did not differ in terms of BMI at the two ages based on a repeated-measures ANOVA (*p* = 0.149). Only 9% of the sample was classified as obese at 18 years of age, while the proportion of obese individuals at 22 years of age reached 16.2%. At 22 years of age, the prevalence of obesity was higher in women than in men (18% versus 13.6%, respectively; *p* < 0.001) (data not shown in tables).

Table 2 shows the evolution of total body and regional adiposity from 18 to 22 years of age, and we can observe that men and women differed in all measurements included in the analysis. On average, women had higher fat mass index and hip and thigh circumferences, while men had higher android fat mass percentages and waist circumferences at both ages. The mean gynoid fat mass was higher in men at 18 years old but higher in women when the individuals were 22 years old.

**Table 2:** Means and differences of total and regional body fatness from 18 to 22 years of age according to sex

	18 years	22 years	p-value^a^	Difference (22 years – 18 years)	p-value^b^
	Mean (95% CI)	Mean (95% CI)		Mean (CI95%)	
*Fat mass index (Kg/m* ^*2*^ *)*			*< 0.001*		*0.196*
Male	4.2 (4.1; 4.4)	5.6 (5.4; 5.7)		1.4 (1.3; 1.5)	
Female	8.4 (8.3; 8.6)	9.9 (9.7; 10.1)		1.5 (1.4; 1.6)	
*Android fat mass (%)*			*< 0.001*		*< 0.001*
Male	8.2 (8.1; 8.3)	9.1 (9.0; 9.2)		1.0 (0.9; 1.0)	
Female	7.6 (7.5; 7.7)	7.8 (7.7; 7.9)		0.2 (0.2; 0.3)	
*Gynoid fat mass (%)*			*< 0.001*		*< 0.001*
Male	21.4 (21.3; 21.6)	20.6 (20.4; 20.7)		-0.9 (−1.0; − 0.8)	
Female	21.2 (21.0; 21.3)	20.9 (20.8; 21.0)		− 0.3 (− 0.4; − 0.2)	
*Waist circumference (cm)*			*< 0.001*		*0.030*
Male	84.5 (84.1; 85.0)	87.0 (86.4; 87.6)		2.7 (2.3; 3.0)	
Female	81.9 (81.5; 82.3)	84.9 (84.3; 85.5)		3.2 (2.9; 3.5)	
*Hip circumference (cm)*			*0.508*		*0.058*
Male	99.4 (99.0; 99.8)	100.9 (100.5; 101.4)		1.6 (1.3; 1.8)	
Female	102.2 (101.8; 102.6)	104.1 (103.6; 104.6)		2.0 (1.7; 2.3)	
*Thigh circumference (cm)*			*0.032*		*0.001*
Male	55.5 (55.3; 55.8)	57.2 (56.9; 57.5)		1.7 (1.5; 1.8)	
Female	56.3 (56.0; 56.6)	58.5 (58.1; 58.8)		2.2 (2.0; 2.5)	

CI95% = 95% confidence interval

^a^p-values are displayed from repeated-measures ANOVA

^b^*p*-values are displayed from the interaction between sex and age in the repeated-measures ANOVA

When we assessed the differences between adiposity measurements from 18 to 22 years of age, we observed that both men and women demonstrated increases in all measurements except for gynoid fat mass percentage. Nevertheless, increases in android fat mass percentage were higher in men, and increases in waist and thigh circumferences were higher in women. We also observed that, on average, both men and women showed a decrease in gynoid fat mass percentage from 18 to 22 years of age. However, this decrease was higher in men than in women (Table 2).

Tables 3 and 4 show differences in total body and regional adiposity from 18 to 22 years of age according to the independent variables included in our study. A higher SEP at 18 years of age was associated with a lower increase in fat mass index and with a lower decrease in gynoid fat mass in women, independent of SEP at birth. In addition, black women had a higher increase in fat mass index (average difference = 1.8 kg/m^2^; 95% CI 1.5 to 2.1) and in android fat mass (average difference = 0.4%; 95% CI 0.2 to 0.5) and a higher decrease in gynoid fat mass (average difference = − 0.5%; 95% CI -0.7 to − 0.3) when compared with white women. In contrast to black women, black men had a lower decrease in gynoid fat mass (mean = − 0.6; 95% CI -0.9 to − 0.4) (Table 3).

**Table 3:** Differences in measures of fat mass from 18 to 22 years of age (measurements at 22 – measurements at 18 years) by sex, according to socioeconomic and demographic characteristics, maternal information and dietary intake at 18 years

	Fat mass index (Kg/m^2^)		Android fat mass (%)		Gynoid fat mass (%)	
	Men	Women	Men	Women	Men	Women
	Mean (95%CI)*	Mean (95%CI)*	Mean (95%CI)*	Mean (95%CI)*	Mean (95%CI)*	Mean (95%CI)*
SEP (quintiles) at 18 years^a^	*0.053* ^*b*^	*0.003* ^*b*^	*0.237* ^*b*^	*0.345* ^*b*^	*0.119* ^*b*^	*0.035* ^*b*^
1stquintile (lowest)	1.0 (0.7; 1.3)	1.6 (1.4; 1.9)	1.0 (0.8; 1.1)	0.2 (0.1; 0.3)	−0.5 (− 0.8; − 0.3)	−0.4 (− 0.5; − 0.2)
2ndquintile	1.4 (1.2; 1.7)	1.7 (1.5; 2.0)	1.1 (1.0; 1.2)	0.3 (0.2; 0.4)	− 1.0 (− 1.3; − 0.8)	−0.5 (− 0.7; − 0.3)
3rdquintile	1.5 (1.2; 1.8)	1.7 (1.4; 1.9)	0.9 (0.8; 1.1)	0.3 (0.2; 0.3)	−0.8 (− 1.1; − 0.6)	−0.4 (− 0.6; − 0.2)
4thquintile	1.5 (1.3; 1.8)	1.5 (1.3; 1.8)	0.9 (0.8; 1.0)	0.3 (0.2; 0.4)	−0.9 (− 1.1; − 0.7)	−0.3 (− 0.5; − 0.1)
5thquintile (highest)	1.5 (1.2; 1.7)	1.0 (0.7; 1.3)	1.0 (0.9; 1.1)	0.1 (0.0; 0.2)	− 1.0 (− 1.2; − 0.8)	−0.1 (− 0.3; 0.1)
Pre-gestational maternal BMI^a^	*0.022* ^*b*^	*0.128* ^*b*^	*0.686* ^*b*^	*0.215* ^*b*^	*0.940* ^*b*^	*0.058* ^*b*^
Normal	1.3 (1.2; 1.5)	1.5 (1.3; 1.6)	1.0 (0.9; 1.1)	0.2 (0.2; 0.3)	−0.9 (− 1.0; − 0.7)	−0.3 (− 0.4; − 0.2)
Overweight	1.7 (1.4; 2.0)	1.7 (1.4; 2.0)	1.0 (0.9; 1.1)	0.3 (0.2; 0.4)	−0.9 (− 1.2; − 0.7)	−0.5 (− 0.7; − 0.3)
Obese	1.7 (1.1; 2.2)	1.7 (1.2; 2.2)	1.0 (0.7; 1.2)	0.3 (0.1; 0.5)	−0.8 (− 1.3; − 0.3)	−0.4 (− 0.8; − 0.1)
Maternal age at birth^a^	*0.916* ^*b*^	*0.924* ^*b*^	*0.148* ^*b*^	*0.249* ^*b*^	*0.577* ^*b*^	*0.897* ^*b*^
18 to 35 years	1.4 (1.3; 1.5)	1.5 (1.4; 1.6)	1.0 (0.9; 1.0)	0.3 (0.2; 0.3)	−0.9 (− 1.0; − 0.7)	−0.3 (− 0.4; − 0.2)
< 18 years	1.4 (1.0; 1.8)	1.7 (1.3; 2.1)	1.1 (0.9; 1.3)	0.2 (0.0; 0.3)	− 1.0 (− 1.4; − 0.7)	−0.5 (− 0.8; − 0.2)
≥ 36 years	1.4 (1.1; 1.8)	1.4 (1.0; 1.8)	1.1 (0.9; 1.3)	0.2 (0.0; 0.3)	− 0.9 (− 1.2; − 0.6)	−0.3 (− 0.5; − 0.1)
Birth weight^a^	*0.252* ^*b*^	*0.439* ^*b*^	*0.248* ^*b*^	*0.475* ^*b*^	*0.762* ^*b*^	*0.153* ^*b*^
< 2500 g	1.5 (1.1; 1.9)	1.4 (1.0; 1.7)	0.9 (0.8; 1.1)	0.2 (0.1; 0.3)	−1.0 (− 1.4; − 0.6)	−0.1 (− 0.3; 0.1)
2500-4000 g	1.4 (1.3; 1.5)	1.5 (1.4; 1.7)	1.0 (0.9; 1.0)	0.2 (0.2; 0.3)	−0.8 (− 1.0; − 0.7)	−0.3 (− 0.4; − 0.3)
> 4000 g	1.9 (1.4; 2.4)	1.6 (1.0; 2.2)	1.1 (0.9; 1.3)	0.2 (0.0; 0.4)	−1.1 (− 1.5; − 0.7)	−0.3 (− 0.7; 0.1)
Skin colour^a^	*0.864* ^*b*^	*0.171* ^*b*^	*0.161* ^*b*^	*0.329* ^*b*^	*0.039* ^*b*^	*0.021* ^*b*^
White	1.4 (1.3; 1.6)	1.4 (1.3; 1.6)	1.0 (0.9; 1.0)	0.2 (0.2; 0.3)	−1.0 (− 1.1; − 0.8)	−0.2 (− 0.3; − 0.1)
Brown	1.5 (1.2; 1.7)	1.6 (1.3; 1.9)	1.1 (1.0; 1.2)	0.2 (0.1; 0.3)	−0.7 (− 1.0; − 0.5)	−0.5 (− 0.6; − 0.3)
Black	1.4 (1.1; 1.7)	1.8 (1.5; 2.1)	1.0 (0.9; 1.1)	0.4 (0.2; 0.5)	−0.6 (− 0.9; − 0.4)	−0.5 (− 0.7; − 0.3)
Daily energy intake in kcal at 18y (quintiles) ^**a**^	*0.297* ^*b*^	*0.376* ^*b*^	*0.556* ^*b*^	*0.014* ^*b*^	*0.564* ^*b*^	*0.081* ^*b*^
1st (lowest)	1.3 (1.1; 1.6)	1.4 (1.3; 1.6)	1.0 (0.9; 1.1)	0.2 (0.1; 0.3)	−0.9 (−1.1; − 0.6)	−0.2 (− 0.3; − 0.1)
2nd	1.4 (1.2; 1.6)	1.5 (1.2; 1.7)	0.9 (0.8; 1.0)	0.2 (0.1; 0.3)	−0.8 (− 1.0; − 0.6)	−0.4 (− 0.5; − 0.2)
3rd (highest)	1.5 (1.3; 1.7)	1.6 (1.4; 1.8)	1.0 (0.9; 1.1)	0.3 (0.2; 0.4)	−0.9 (− 1.1; − 0.8)	−0.4 (− 0.5; − 0.2)

*Mean displayed for the difference between measure collected at 22 - measure collected at 18)

^a^All analyses were adjusted for SEP at birth; ^b^p-values are displayed from linear regression

**Table 4:** Differences in body circumferences from 18 to 22 years of age (measurements at 22 – measurements at 18 years) by sex, according to socioeconomic and demographic characteristics, maternal information and dietary intake at 18 years

	Waist circumference (cm)		Hip circumference (cm)		Thigh circumference (cm)	
	Men	Women	Men	Women	Men	Women
	Mean (95%CI)*	Mean (95%CI)*	Mean (95%CI)*	Mean (95%CI)*	Mean (95%CI)*	Mean (95%CI)*
SEP (quintiles)^a^	*0.187* ^*b*^	*0.001* ^*b*^	*0.270* ^*b*^	*0.003* ^*b*^	*0.142* ^*b*^	*0.004* ^*b*^
1^st^quintile (lowest)	1.8 (1.0; 2.6)	3.6 (2.9; 4.3)	1.0 (0.3; 1.6)	2.3 (1.8; 2.9)	1.2 (0.8; 1.6)	2.7 (2.2; 3.1)
2^nd^quintile	2.7 (2.0; 3.5)	4.0 (3.2; 4.8)	1.7 (1.1; 2.2)	2.4 (1.8; 3.0)	1.6 (1.2; 1.9)	2.4 (1.9; 2.9)
3^rd^quintile	3.0 (2.3; 3.7)	3.5 (2.8; 4.2)	1.7 (1.2; 2.3)	2.5 (1.9; 3.1)	2.0 (1.6; 2.4)	2.6 (2.2; 3.1)
4^th^ quintile	2.8 (2.1; 3.5)	3.0 (2.2; 3.7)	1.7 (1.1; 2.2)	1.7 (1.1; 2.4)	1.6 (1.3; 2.0)	2.0 (1.5; 2.5)
5^th^ quintile (highest)	2.7 (2.0; 3.4)	1.8 (0.9; 2.6)	1.6 (1.0; 2.2)	1.0 (0.3; 1.6)	1.7 (1.3; 2.1)	1.6 (1.0; 2.1)
Pre-gestational maternal BMI ^a^	*0.177* ^*b*^	*0.040* ^*b*^	*0.839* ^*b*^	*0.422* ^*b*^	*0.413* ^*b*^	*0.361* ^*b*^
Normal	2.5 (2.2; 2.9)	3.0 (2.6; 3.4)	1.5 (1.2; 1.8)	1.9 (1.6; 2.2)	1.6 (1.4; 1.8)	2.2 (2.0; 2.4)
Overweight	3.4 (2.6; 4.1)	3.9 (3.1; 4.7)	2.0 (1.4; 2.6)	2.5 (1.8; 3.1)	1.8 (1.4; 2.2)	2.5 (2.0; 3.0)
Obese	2.7 (1.2; 4.1)	3.9 (2.4; 5.4)	0.9 (−0.2; 2.1)	1.8 (0.6; 3.0)	1.7 (1.0; 2.5)	2.3 (1.4; 3.3)
Maternal age at birth ^a^	*0.143* ^*b*^	*0.902* ^*b*^	*0.153* ^*b*^	*0.867* ^*b*^	*0.040* ^*b*^	*0.769* ^*b*^
18 to 35 years	2.8 (2.4; 3.1)	3.2 (2.8; 3.5)	1.7 (1.4; 1.9)	2.0 (1.7; 2.3)	1.7 (1.5; 1.9)	2.3 (2.0; 2.5)
< 18 years	2.4 (1.3; 3.6)	3.5 (2.3; 4.8)	1.5 (0.5; 2.4)	2.3 (1.3; 3.2)	1.5 (0.9; 2.1)	2.8 (2.0; 3.6)
≥ 36 years	2.0 (1.0; 3.0)	3.1 (2.0; 4.3)	1.0 (0.2; 1.8)	1.8 (0.9; 2.7)	1.1 (0.6; 1.7)	1.9 (1.2; 2.6)
Birth weight ^a^	*0.732* ^*b*^	*0.349* ^*b*^	*0.427* ^*b*^	*0.537* ^*b*^	*0.929* ^*b*^	*0.393* ^*b*^
< 2500 g	2.8 (1.6; 4.0)	2.7 (1.6; 3.7)	2.0 (1.0; 2.9)	1.6 (0.7; 2.4)	1.8 (1.2; 2.4)	1.7 (1.1; 2.3)
2500-4000 g	2.6 (2.3; 3.0)	3.3 (2.9; 3.6)	1.6 (1.3; 1.8)	2.1 (1.8; 2.4)	1.6 (1.5; 1.8)	2.4 (2.2; 2.6)
> 4000 g	3.1 (1.9; 4.4)	3.3 (1.6; 5.0)	1.4 (0.4; 2.4)	1.5 (0.2; 2.9)	1.8 (1.1; 2.4)	1.5 (0.5; 2.7)
Skin colour ^a^	*0.879* ^*b*^	*0.076* ^*b*^	*0.543* ^*b*^	*0.708* ^*b*^	*0.502* ^*b*^	*0.207* ^*b*^
White	2.7 (2.3; 3.1)	2.8 (2.4; 3.3)	1.5 (1.2; 1.9)	1.9 (1.6; 2.3)	1.6 (1.4; 1.8)	2.1 (1.8; 2.4)
Brown	2.8 (2.0; 3.6)	3.4 (2.6; 4.2)	1.8 (1.1; 2.4)	2.0 (1.4; 2.7)	1.7 (1.3; 2.2)	2.4 (1.9; 2.9)
Black	2.6 (1.7; 3.4)	4.3 (3.4; 5.2)	1.5 (0.8; 2.2)	2.2 (1.5; 2.9)	1.8 (1.4; 2.3)	2.7 (2.1; 3.2)
Daily energy intake in kcal at 18y (quintiles) ^a^	*0.890* ^*b*^	*0.673* ^*b*^	*0.168* ^*b*^	*0.910* ^*b*^	*0.296* ^*b*^	*0.336* ^*b*^
1^st^ (lowest)	2.7 (2.0; 3.3)	3.1 (2.5; 3.7)	1.4 (0.9; 1.9)	2.1 (1.7; 2.5)	1.6 (1.2; 1.9)	2.2 (1.8; 2.6)
2^nd^	2.8 (2.2; 3.3)	2.9 (2.3; 3.5)	1.6 (1.1; 2.0)	1.6 (1.1; 2.1)	1.7 (1.4; 2.0)	2.1 (1.7; 2.5)
3^rd^ (highest)	2.7 (2.2; 3.2)	3.3 (2.7; 4.0)	1.9 (1.4; 2.3)	2.2 (1.7; 2.7)	1.8 (1.5; 2.1)	2.5 (2.1; 2.9)

*Mean displayed for the difference between measure collected at 22 - measure collected at 18)

^a^All analyses were adjusted for SEP at birth; ^b^p-trends from linear regression are displayed

When we assessed differences in body circumferences according to independent variables, we observed that SEP at 18 years of age was negatively associated with differences in female measures of waist, hip and thigh circumferences. The most affluent women (classified in the 5th quintile of SEP at 18 years of age) showed a lower increase in waist circumference (average difference = 1.8 cm; 95% CI 0.9 to 2.6), hip circumference (average difference = 1.0 cm; 95% CI 0.3 to 1.6) and thigh circumference (average difference = 1.6 cm; 95% CI 1.0 to 2.1) from 18 to 22 years of age. Moreover, men whose mothers were > 35 years old by the time of birth showed a lower increase in thigh circumference (average difference = 1.1; 95% CI 0.6 to 1.7). Finally, black women showed a higher increase in waist circumference from 18 to 22 years of age when compared to white women, independent of SEP at birth (average difference = 4.3 cm; 95% CI 3.4 to 5.2) (Table 4).

## Discussion

Our study assessed the evolution of total body and regional adiposity measured by DXA and a 3-D Photonic Scanner from late adolescence to early adulthood in a population-based Brazilian sample. In Brazil, recent nationally representative studies have shown an increase in obesity prevalence in children and adolescents [21]. Our results were not different from the Brazilian national results, as both men and women demonstrated increases in BMI, with means above 25 kg/m^2^ at 22 years of age. In addition, the obesity prevalence in this cohort rose from less than 10 to 16% in four years.

As we expected, women had a higher fat mass index than men, despite no differences in increases of fat mass index according to sex. Nevertheless, the gain of 1.4 kg/m^2^ in men’s fat mass index represented an increase of 35 percentage points, while the absolute gain in women’s fat mass index (1.5 kg/m^2^) represented an increase of only 10 percentage points (*p* < 0.001).

We also observed that despite both men and women having a gain in all measurements except for gynoid fat mass percentage, men demonstrated higher increases in android fat mass percentage from 18 to 22 years of age and higher decreases in gynoid fat mass when compared to women. This result suggests that men had greater centralization of adiposity from 18 to 22 years of age, resulting in a more android body shape.

Adolescence is a period where sex differences in body shape increase and the relative proportion of fat tissue starts to differ substantially [15]. Early adulthood is marked by the largest difference in body composition and shape between men and women, reflecting sex-hormones’ actions on the distribution of body fat [22]. Previous studies have reported a more centralized body shape in men after the onset of puberty, while women present higher proportions of peripheral fat mass [22, 23]. These differences in body fat distribution, which were reinforced by our results, are important in the obesity-disease relationship, since android body shape is more strongly associated with disease risks and mortality [8, 24], while gynoid body shape is more strongly linked to beneficial metabolic traits [7, 10].

Another notable finding of our study was the social patterning of total body and regional adiposity in this Brazilian sample. Our results showed that changes in body composition from 18 to 22 years of age were strongly associated with SEP in women. Women in the highest quintile of SEP had lower increases in measurements of android body shape from adolescence to early adulthood. In Brazil, nationally representative investigations have shown that SEP is positively associated with overweight and obesity in children and adult men and negatively associated in women [4]. A study using data from the 1982 and 1993 Pelotas cohort studies found a positive association between SEP and obesity prevalence in 11-year-old boys and girls from the 1993 cohort and a negative association in 18-year-old women from the 1982 cohort [25]. The lower increase in central measurements from 18 to 22 years of age among the most affluent women might indicate the life stage where the association between SEP and adiposity becomes inversed in women.

We also revealed a relationship between adiposity and race, independent of SEP at birth. Wells et al. [26] described that African Americans have larger circumferences than Caucasians. Despite the lack of cross-sectional associations of adiposity measurements with race at 18 and 22 years of age, we observed that black women showed higher increases in fat mass index and greater centralization of body shape from 18 to 22 years of age, which may increase their risk of noncommunicable diseases in the future.

Surprisingly, daily energy intake at 18 years of age was poorly associated with the distribution of total body and regional adiposity from 18 to 22 years of age after adjustment for SEP at birth. It is important to highlight that we did not take energy expenditure into account in this analysis, and daily energy intake is related to energy expenditure. Nevertheless, Goldberg et al. [27] showed a strong association between energy intake and energy expenditure in stable weight periods. Literature has shown that associations between dietary energy intake and fat mass are quite controversial [28, 29], and a recent study showed that daily energy intake is associated with lean mass but not with fat mass or BMI in obese adults [30].

The repeated body composition and body shape assessments, allowing us to take not only overall body mass into account but also the distribution of body fat, may be considered a strength of our study. The use of two different approaches to measure total body and regional adiposity was a further strength. In addition, regular data collection with high retention rates was an important factor to minimize biases. Limitations included the lack of adjustment for physical activity in statistical analyses, since physical activity may serve a key role in the evolution of total body and regional adiposity from late adolescence to early adulthood.

## Conclusions

In conclusion, our study showed an increase in total body and regional adiposity as well as a centralization of body shape from late adolescence to early adulthood in both men and women using population-based data from a middle-income country. From the clinical and public health points of view, these results are alarming because high levels of total body and regional adiposity in early stages of life may put this population at high risk for diseases and mortality in the short- and long-term.

## Abbreviations

ANOVA: analysis of variance
BMI: body mass index
DXA: dual x-ray absorptiometry
FFQ: food frequency questionnaire
SEP: socioeconomic position
UK: United Kingdom
USA: United States of America
WHO: World Health Organization
